# Eravacycline, the first four years: health outcomes and tolerability data for 19 hospitals in 5 U.S. regions from 2018 to 2022

**DOI:** 10.1128/spectrum.02351-23

**Published:** 2023-11-29

**Authors:** Ashlan J. Kunz Coyne, Sara Alosaimy, Kristen Lucas, Abdalhamid M. Lagnf, Taylor Morrisette, Kyle C. Molina, Alaina DeKerlegand, Melanie Rae Schrack, S. Lena Kang-Birken, Athena L.V. Hobbs, Jazmin Agee, Nicholson B. Perkins, Mark Biagi, Michael Pierce, James Truong, Justin Andrade, Jeannette Bouchard, Tristan Gore, Madeline A. King, Benjamin M. Pullinger, Kimberly C. Claeys, Shelbye Herbin, Reese Cosimi, Serina Tart, Michael P. Veve, Bruce M. Jones, Leonor M. Rojas, Amy K. Feehan, Marco R. Scipione, Jing J. Zhao, Paige Witucki, Michael J. Rybak

**Affiliations:** 1 Department of Pharmacy Practice, Anti-Infective Research Laboratory, Eugene Applebaum College of Pharmacy and Health Sciences, Wayne State University, Detroit, Michigan, USA; 2 Department of Emergency Medicine, University of Colorado School of Medicine, Aurora, Colorado, USA; 3 Our Lady of the Lake Regional Medical Center, Baton Rouge, Louisiana, USA; 4 Santa Barbara Cottage Hospital, Santa Barbara, California, USA; 5 Methodist University Hospital, Memphis, Tennessee, USA; 6 UW Health SwedishAmerican Hospital, Rockford, Illinois, USA; 7 University of Illinois at Chicago, Rockford, Illinois, USA; 8 NewYork-Presbyterian Hospital, Queens, New York, USA; 9 Touro College of Pharmacy, The Brooklyn Hospital Center, New York, New York, USA; 10 College of Pharmacy, University of South Carolina, Columbia, South Carolina, USA; 11 Philadelphia College of Pharmacy, Saint Joseph’s University, Philadelphia, Pennsylvania, USA; 12 Cooper University Hospital, Camden, New Jersey, USA; 13 University of Maryland School of Pharmacy, Baltimore, Maryland, USA; 14 Department of Pharmacy, Henry Ford Hospital, Detroit, Michigan, USA; 15 Ascension St. Vincent Hospital, Indianapolis, Indiana, USA; 16 Cape Fear Valley Health, Fayetteville, North Carolina, USA; 17 University of Tennessee Health Science Center College of Pharmacy, Memphis, Tennessee, USA; 18 University of Tennessee Medical Center, Knoxville, Tennessee, USA; 19 St. Joseph’s/Candler Health System, Savannah, Georgia, USA; 20 Valley Hospital Medical Center, Las Vegas, Nevada, USA; 21 Ochsner Clinic Foundation, New Orleans, Louisiana, USA; 22 Ochsner Clinical School, The University of Queensland, New Orleans, Louisiana, USA; 23 Department of Pharmacy, Detroit Receiving Hospital, Detroit Medical Center, Detroit, Michigan, USA; 24 Department of Pharmacy, Harper University Hospital, Detroit Medical Center, Detroit, Michigan, USA; 25 Department of Medicine, Division of Infectious Diseases, School of Medicine, Wayne State University, Detroit, Michigan, USA; Emory University School of Medicine, Atlanta, Georgia, USA

**Keywords:** eravacycline, multidrug-resistant, antimicrobial stewardship

## Abstract

Eravacycline is a synthetic fluorocycline approved by the U.S. Food and Drug Administration in 2018. This study aimed to describe clinical and microbiological outcomes in addition to associated adverse effects of eravacycline used in U.S. hospitals. Real-world, observational study involving patients receiving ≥72 h of eravacycline at 19 medical centers located in all 5 regions of the United States between October 2018 and August 2022. The primary outcome was clinical success, defined as survival and absence of microbiological recurrence at 30 days from the end of eravacycline therapy and clinical improvement within 96 h of eravacycline initiation. In total, 416 patients met study criteria and were evaluated. Index culture specimens were most often isolated from the respiratory tract (24.8%, *n* = 103/416), wound(s) (20.9%, *n* = 87/416), or blood (19.5%, *n* = 81/416). As definitive therapy, eravacycline was most often used to treat infections caused by *Enterobacterales* spp. (42.3%, *n* = 176/416; 24.4%, *n* = 43/176 carbapenem-resistant), *Enterococci* spp. (24.0%, *n* = 100/416; 49.0%, 49/100 vancomycin-resistant), and *Acinetobacter* spp. (23.3%, *n* = 97/416; 47.4%, *n* = 46/97 carbapenem-resistant). Clinical success occurred in 75.7% of patients (*n* = 315/416). Thirty-nine (9.4%, *n* = 39/416) patients experienced a treatment emergent adverse event (TEAE) potentially related to eravacycline with the majority (51.3%, *n* = 20/39) being gastrointestinal intolerance. Only 27 isolates (6.5%, *n* = 27/416) underwent eravacycline susceptibility testing. Eravacycline is being used to treat a broad range of Gram-negative and Gram-positive bacteria in the United States including those demonstrating multidrug-resistance with consistently low reported drug-related TEAE; however, antimicrobial susceptibility testing and subsequent *in vitro* susceptibility data of clinical isolates was sparingly performed.

**IMPORTANCE:**

The rise of multidrug-resistant (MDR) pathogens, especially MDR Gram-negatives, poses a significant challenge to clinicians and public health. These resilient bacteria have rendered many traditional antibiotics ineffective, underscoring the urgency for innovative therapeutic solutions. Eravacycline, a broad-spectrum fluorocycline tetracycline antibiotic approved by the FDA in 2018, emerges as a promising candidate, exhibiting potential against a diverse array of MDR bacteria, including Gram-negative, Gram-positive, anaerobic strains, and Mycobacterium. However, comprehensive data on its real-world application remain scarce. This retrospective cohort study, one of the largest of its kind, delves into the utilization of eravacycline across various infectious conditions in the USA during its initial 4 years post-FDA approval. Through assessing clinical, microbiological, and tolerability outcomes, the research offers pivotal insights into eravacycline’s efficacy in addressing the pressing global challenge of MDR bacterial infections.

## INTRODUCTION

Faced with the escalating challenge of antimicrobial resistance, which threatens an estimated 10 million lives annually by 2050 due to the spread of multidrug-resistant (MDR) pathogens, novel solutions are imperative (1 – 3). In this context, the U.S. Food and Drug Administration (FDA) approved eravacycline in August 2018 for treating complicated intraabdominal infections (cIAI) (4). As the first fully synthetic fluorocycline, eravacycline maintains stability against the efflux pumps and ribosomal protection proteins that typically confer resistance to other members of the tetracycline antibiotic class (5). Eravacycline has shown potent *in vitro* activity against a wide range of Gram-negative bacteria, including carbapenem-resistant isolates. This includes bacteria such as Enterobacterales that produce an extended-spectrum beta-lactamase or carbapenemase, *Acinetobacter* species, and other MDR Gram-negative pathogens. Several studies have reported low minimum inhibitory concentration (MIC_90_) values for eravacycline against these bacteria (6 – 10). Additionally, eravacycline has demonstrated activity *in vitro* against Gram-positive bacteria including methicillin-resistant *Staphylococcus aureus* (MRSA) and vancomycin-resistant *Enterococcus* (VRE), nontuberculosis mycobacteria, and anaerobic bacteria such as *Clostridioides difficile* (11 – 13).

The efficacy of eravacycline in treating cIAIs was established through two phase III multicenter clinical randomized controlled trials (RCTs) (IGNITE I/IV), demonstrating noninferiority to ertapenem and meropenem, respectively (14, 15). Furthermore, real-world experience with eravacycline, although limited, has shown comparable clinical success and tolerability to the clinical trials (16). Retrospective studies utilizing real-world data provide valuable insights into real-world outcomes and usage, complementing RCTs by exploring long-term outcomes, rare adverse events, and complex relationships in diverse populations. The objective of this study is to describe the clinical use of eravacycline in United States hospitals in terms of clinical and microbiological response and drug-related adverse events in its first 4 years following FDA approval.

## MATERIALS AND METHODS

Retrospective cohort study using inpatient data from October 2018 to August 2022 of adult patients admitted to a participating medical center and receipt of ≥72 consecutive hours of eravacycline therapy for any pathogen within the spectrum of eravacycline activity isolated from any infectious source. Participating centers encompassed a diverse range of medical institutions including academic/university-affiliated centers and community hospitals situated in both urban and suburban locales. Exclusion criteria included patients who were pregnant or nursing, prisoners, and those that received subsequent eravacycline courses not separated by at least 90 days from the end of the index eravacycline treatment course.

The primary outcome was clinical success, defined as survival with absence of microbiological recurrence at 30 days from the end of eravacycline therapy and clinical improvement within 96 hours of eravacycline initiation. Microbiological recurrence was defined as a positive culture for the same organism and infectious source within 30 days from the end of eravacycline therapy. Clinical improvement was defined as the resolution of infectious signs and symptoms including infection-related abnormal white blood cell count/temperature or as documented by the physician in clinical notes. Key secondary clinical, microbiological, and tolerability endpoints including hospital readmission, infection-related readmission, and possible eravacycline-related adverse effects using the common terminology criteria for adverse events were also evaluated (17). The relationship of possible treatment emergent adverse events (TEAEs) related to eravacycline was determined based on adverse event onset in relation to the initiation and possible discontinuation of eravacycline using medical record documentation. Concomitant therapy was defined as any therapy used in conjunction with eravacycline for ≥48 continuous hours for the primary organism that eravacycline therapy was used for.

To obtain information on patient demographics and baseline characteristics, we accessed the electronic health record (EHR) and recorded the data in Research Electronic Data Capture (REDCap) (18). The Charlson Comorbidity Index was used to estimate comorbidity burden, while measures of organ function and illness severity were assessed based on the highest Acute Physiology and Chronic Health Evaluation II (APACHE II) and Sequential Organ Failure Assessment (SOFA) score within 48 h of index culture collection. Index culture was defined as the culture collected closest to eravacycline initiation. Immunosuppressive factors were defined as neutropenia (absolute neutrophil count <500), splenectomy (functional or surgical), or high dose corticosteroids (>prednisone 20 mg/day or equivalent). All cultures, bacterial identifications, and antibiotic susceptibilities were conducted according to local procedures at each center. Clinical Laboratory Standards Institute (CLSI) breakpoints were used to interpret MIC results, where applicable (19).

Descriptive statistics were employed to evaluate baseline characteristics. Frequencies and percentages were used to report discrete data, while continuous data were described using median and interquartile range (IQR) or mean and standard deviation (SD), depending on the normality of the distribution. IBM SPSS Statistics version 29 (IBM Corp., Armonk, NY) was used to carry out the analyses.

## RESULTS

### Demographics

A total of 416 patients receiving ≥72 h of eravacycline were included from 19 medical centers located in all five geographic regions of the United States. Baseline demographic data are displayed in Table 1. The mean (SD) age was 58.7 (15) years, and most patients were male (56.7%, *n* = 236/416), Caucasian (56.7%, *n* = 236/416), and admitted from home (57.2%, *n* = 238/416). The median (IQR) Charlson Comorbidity Index was 4.5 (2, 7) with diabetes (36.3%, *n* = 151/416), heart failure (18.3%, *n* = 76/416), and peripheral vascular disease (18%, *n* = 75/416) being the most common comorbid conditions. At least one immunosuppressive factor was identified in 16.6% (*n* = 69/416) of patients and 87.2% (*n* = 363/416) of patients had at least one MDR risk factor with the most common being ≥48 h hospitalization (55.3%, *n* = 230/416) and ≥24 h antibiotics (53.4%, *n* = 222/416) within 90 days prior to index positive culture collection for which eravacycline was used as definitive therapy.

**TABLE 1:** Baseline characteristics (*n* = 416)^*a*^ ^,^ ^*c*^ ^,^ ^*d*^

Parameter	Value
Age (mean) (years) ± SD	58.7 ± 15
Male	236 (56.7)
Race	
African American	122 (29.3)
Asian	9 (2.2)
Caucasian	236 (56.7)
Hispanic	41 (9.9)
Other	5 (1.2)
BMI (kg/m^2^)	26.4 (22.6, 32.4)
Obese (BMI ≥30 kg/m^2^)	133 (32)
Admitted from	
Home	238 (57.2)
NH/LTC	80 (19.2)
Transfer from outside hospital	61 (14.7)
Other	37 (8.9)
Severity scores	
APACHE II score	16 (11, 25)
SOFA score	4.5 (2.5, 7)
Charlson Comorbidity Index	4.5 (2, 7)
Comorbid conditions	
Heart failure	76 (18.3)
COPD	65 (15.6)
Diabetes	151 (36.3)
Chronic kidney disease	71 (17.1)
HD dependent	30 (7.2)
*Clostridiodes difficile^b^*	27 (6.5)
Immunosuppression factors	
Neutropenia	15 (3.6)
AIDS	1 (0.2)
Splenectomy	6 (1.4)
Solid organ transplant	10 (2.4)
Bone marrow transplant	3 (0.7)
Cytotoxic chemotherapy	22 (5.3)
High dose corticosteroids	12 (2.9)
MDR risk factors	
≥24 h antibiotics within 90 days	222 (53.4)
≥48 h hospitalization within 90 days	230 (55.3)
NH/LTC resident	80 (19.2)
Home infusion	18 (4.3)
Chronic dialysis	24 (5.8)
Home wound care	38 (9.1)
Surgery ≤30 days before index culture	75 (18)
Colonization with resistant organism(s)	46 (11.1)
Prior infection with resistant organism	104 (25)

^*a*^ Data presented as number (%) or median (IQR), as appropriate.

^*b*^ History of *Clostridiodes difficile* infection.

^*c*^ Immunosupression factors: neutropenia (absolute neutrophil count <500); splenectomy (functional or surgical); high dose corticosteroids (≥ prednisone 20 mg/day or equivalent).

^*d*^ SD, standard deviation; BMI, body mass index; LTAC, long-term acute care; NH/LTC, nursing home/long-term care facility; APACHE II, Acute Physiology and Chronic Health Evaluation II; SOFA, sequential organ failure assessment; COPD, chronic obstructive pulmonary disease; HD, hemodialysis; HIV, human immunodeficiency virus; IVDU, intravenous drug user; AIDS, acute immunodeficiency syndrome.

### Clinical course and treatment characteristics

**Fig 1:**
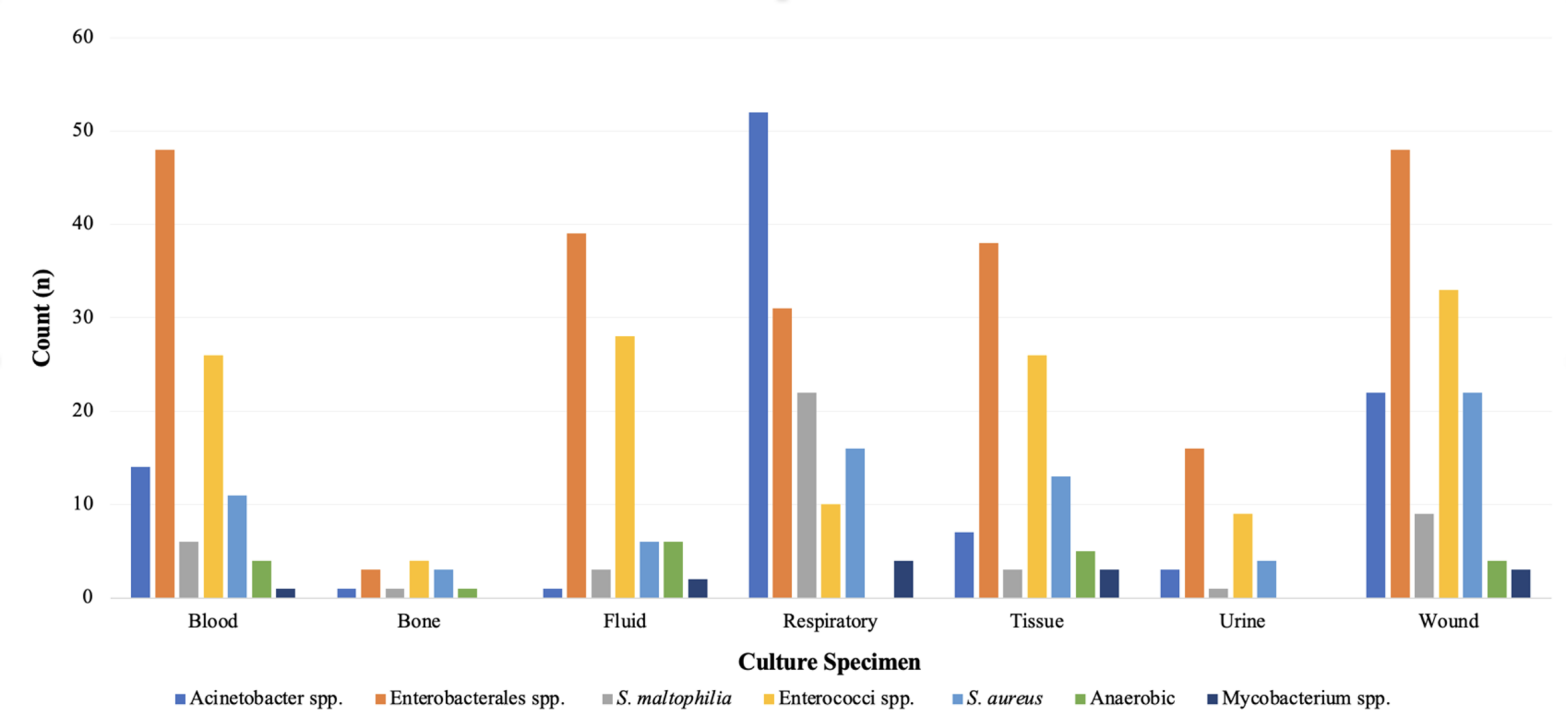
Microbiological isolates and culture specimen source.

**Fig 2:**
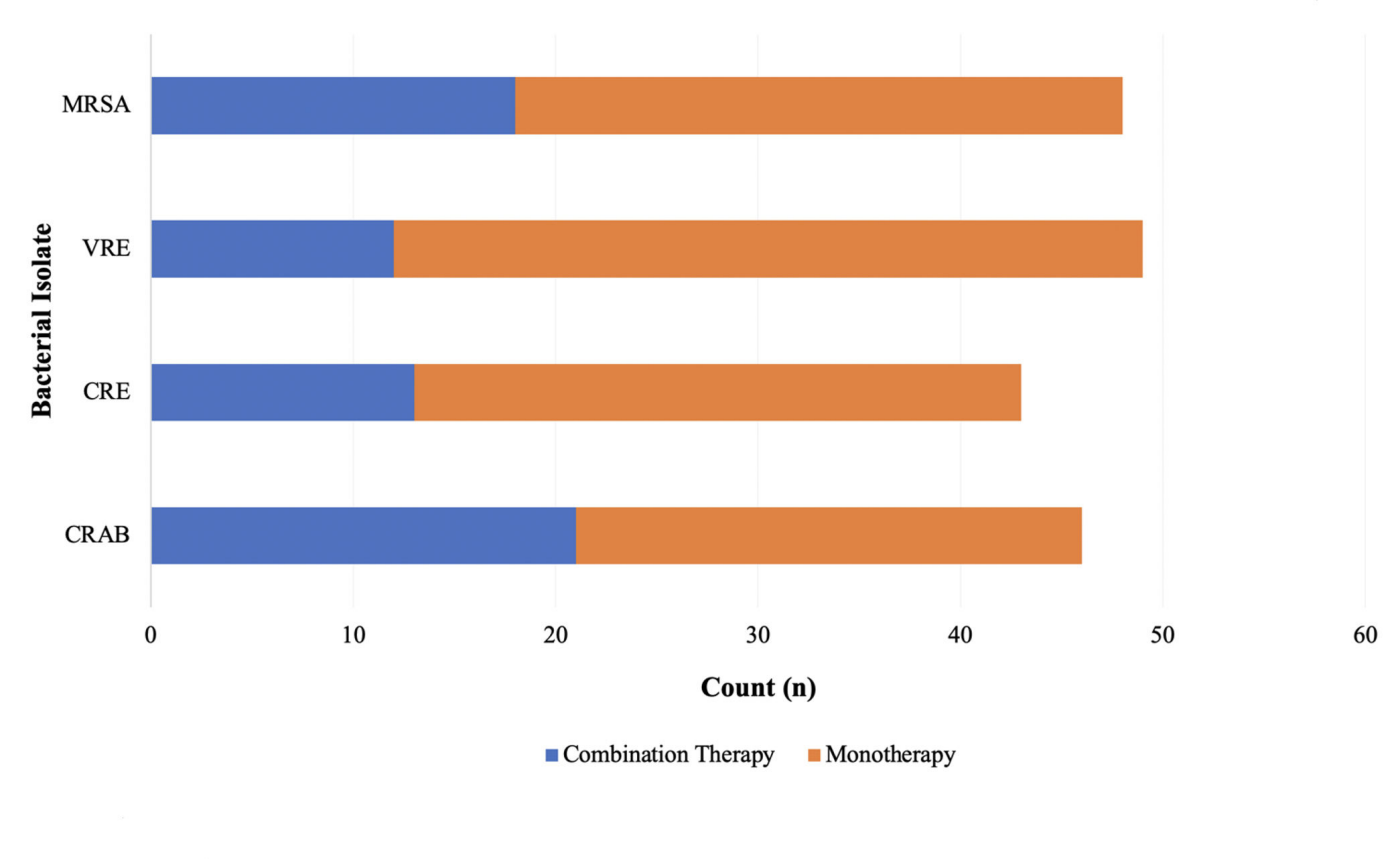
Use of combination therapy versus monotherapy for resistant bacterial isolates. Abbreviations: MRSA, methicillin-resistant *Staphylococcus aureus*; VRE, vancomycin-resistant enterococci; CRE, carbapenem-resistant Enterobacterales; CRAB, carbapenem-resistant *Acinetobacter baumannii*.

Clinical course and treatment characteristics are displayed in Table 2. In total, 42.5% (*n* = 177/416) of patients were admitted to the intensive care unit (ICU) at least once during the hospital admission with 26.9% (*n* = 112/416) being in the ICU at the time of index culture collection. Infections treated with eravacycline were classified as hospital-acquired in 49.5% (*n* = 206/416) of cases with a median (IQR) of 3 (1, 14) days from hospital admission to index culture collection. Index culture specimens were most often isolated from the respiratory tract (24.8%, *n* = 103/416), wound(s) (20.9%, *n* = 87/416), or blood (19.5%, *n* = 81/416). Figure 1 displays microbiological characteristics of index culture specimens. Infectious diseases and/or surgical consultations were initiated in 91.3% (*n* = 380/416) and 51.4% (*n* = 214/416) of patients, respectively. More than one-half of patients underwent a surgical procedure for source control (52.6%, *n* = 219/416) with incision and drainage (16.3%, *n* = 68/416) and debridement (12.3%, *n* = 51/416) being the most common procedures. Receipt of antimicrobial therapy with *in vitro* activity prior to the initiation of eravacycline occurred in 47.1% (*n* = 196/416) of patients and most often consisted of a carbapenem (29.6%, *n* = 58/196) or aminoglycoside (11.2%, *n* = 22/196) for isolated Gram-negative bacteria and vancomycin (16.8%, *n* = 70/196) or linezolid (12.8%, *n* = 25/196) for Gram-positive bacteria. The median (IQR) time from index culture collection to the first administration of eravacycline was 4 (2, 8) days. Consolidation of the antibiotic regimen was the most common reason for selecting eravacycline as definitive therapy (39.7%, *n* = 165/416). Most patients received eravacycline according to the package insert-recommended dosing of 1 mg/kg (96.9%, *n* = 403/416) administered every 12 h (95%, *n* = 395/416). The median duration of eravacycline therapy was 6.9 (4.1, 11.9) days. Approximately one-half of patients (50.7%, *n* = 211/416) received ≥48 h of concomitant antibiotic therapy with eravacycline, which was most often either meropenem (17.5%, *n* = 37/211) or amikacin (8.5%, *n* = 18/211). Figure 2 displays the use of combination therapy versus monotherapy for the treatment of select resistant bacterial isolates.

**TABLE 2:** Clinical course and treatment characteristics (*n* = 416)^*a*^ ^,^ ^*f*^ ^,^ ^*g*^

Parameter	Value
ICU admissions	
At least one ICU admission during hospitalization	177 (42.5)
Patients with >1 ICU admission during hospitalization	43 (10.3)
In ICU at index culture collection	112 (26.9)
ICU length of stay (days)	14 (7, 32)
Mechanical ventilation for ≥48 h prior to index positive culture	68 (16.3)
Duration of mechanical ventilation (days)	22.5 (12, 36)
Culture information	
Hospital-acquired infection^*b*^	206 (49.5)
Culture specimen	
Respiratory culture specimen	103 (24.8)
Aspirate	20 (4.8)
Bronchoalveolar lavage	15 (3.6)
Sputum	68 (16.3)
Wound	87 (20.9)
Blood	81 (19.5)
Fluid	57 (13.7)
Tissue	53 (12.7)
Urine	17 (4.1)
Fecal	13 (3.1)
Bone	8 (1.9)
Catheter tip	2 (0.5)
Concomitant bacteremia	8 (1.9)
ID consult	380 (91.3)
Surgery consult	214 (51.4)
Surgical intervention^*c*^	219 (52.6)
Active therapy before ERV^*d*^	196 (47.1)
Aminoglycoside	22 (11.2)
Carbapenem	58 (29.6)
Cefepime	44 (10.6)
Ceftazidime-avibactam	16 (3.8)
Ceftolozane-tazobactam	2 (0.5)
Meropenem-vaborbactam	3 (0.7)
Polymyxins	2 (1)
Quinolone	11 (5.6)
Tigecycline	8 (1.9)
Trimethoprim/sulfamethoxazole	10 (2.4)
Daptomycin	23 (5.5)
Linezolid/tedizolid	25 (12.8)
Vancomycin	70 (16.8)
Other	72 (36.7)
ERV treatment specifics	
Rationale for ERV use	
Consolidation of regimen	165 (39.7)
Lack of oral access	16 (3.8)
Double CRE coverage	31 (7.5)
ERV regimens	
Dose	
1 mg/kg	403 (96.9)
1.5 mg/kg	13 (3.1)
Frequency	
Every 12 h	395 (95)
Every 12 h on day 1, then every 24 h	14 (3.3)
Every 24 h	7 (1.7)
ERV duration of therapy	6.9 (4.1, 11.9)
Concomitant therapy^*e*^	211 (50.7)
Amikacin	18 (4.3)
Aztreonam	2 (0.5)
Ciprofloxacin	5 (1.2)
Colistin	5 (1.2)
Ertapenem	2 (0.5)
Gentamicin	1 (0.2)
Imipenem	5 (1.2)
Levofloxacin	7 (1.7)
Meropenem	37 (8.9)
Polymyxin B	6 (1.4)
TMP/SMX	9 (2.2)
Tobramycin	9 (2.2)
Other	111 (26.7)
Discharge disposition	
Home	154 (37)
LTAC	108 (26)
Rehab center	35 (8.4)
Outside hospital	1 (0.2)
Hospice	28 (6.7)
Morgue	82 (19.7)
Hospital length of stay (days)	21 (11, 41)

^*a*^ Data presented as number (%) or median (IQR), as appropriate.

^*b*^ Hospital-acquired infection: Index positive culture collected ≥48 h from hospital admission (includes time accrued at previous institution if the patient transferred from an outside hospital).

^*c*^ Surgical intervention: Incision and drainage (*n* = 68), debridement (*n* = 51), amputation (*n* = 10), valvular replacement (*n* = 2), invasive device removal (*n* = 6), other (*n* = 82).

^*d*^ Total may exceed *n* of 416 due to receipt of multiple antibiotics.

^*e*^ Active therapy: Demonstrated *in vitro* susceptibility.

^*f*^ Concomitant therapy: Antibiotic administered for ≥48 continuous hours while the patient received eravacycline.

^*g*^ ICU, intensive care units; ID, infectious diseases; ERV, eravacycline; CRE, carbapenem-resistant Enterobacterales; LTAC, long-term acute care.

### Microbiological characteristics

All isolated organisms and those for which eravacycline was used as definitive therapy are displayed in Table 3. Eravacycline was most often ordered as definitive therapy to treat infections caused by *Acinetobacter* spp. [23.3%, *n* = 97/416; 47.4% of which were carbapenem-resistant *Acinetobacter* (*n* = 46/97)] or *Enterococci* spp. [24%, *n* = 100/416; 49% (*n* = 49/100) of which were vancomycin-resistant Enterococci]. Eravacycline was also often used as definitive therapy for carbapenem-resistant Enterobacterales (CRE) (10.3%, *n* = 43/416) and *Stenotrophomonas maltophilia* (9.9%, *n* = 41/416).

**TABLE 3:** Definitive eravacycline therapy

Parameter	Value
Gram-negative	
*Achromobacter* spp.	4 (1)
*Acinetobacter* spp.	97 (23.3)
*Acinetobacter baumannii*	92 (22.1)
Carbapenem-resistant *Acinetobacter* spp.	46 (11.1)
Enterobacterales	176 (42.3)
*Citrobacter freundii*	6 (1.4)
*Enterobacter cloacae*	33 (7.9)
*Escherichia coli*	50 (12)
*Klebsiella aerogenes*	5 (1.2)
*Klebsiella oxytoca*	12 (2.9)
*Klebsiella pneumoniae*	54 (13)
*Morganella morganii*	4 (1)
*Proteus mirabilis*	5 (1.2)
*Proteus vulgaris*	1 (0.2)
*Providencia stuartii*	3 (0.7)
*Serratia marcescens*	3 (0.7)
Carbapenem-resistant Enterobacterales	43 (10.3)
*Pseudomonas aeruginosa*	0 (0)
*Stenotrophomonas maltophilia*	41 (9.9)
Gram-positive	
Enterococci	100 (24)
*Enterococcus faecalis*	45 (10.8)
*Enterococcus faecium*	55 (13.2)
Vancomycin-resistant enterococci	49 (11.8)
*Staphylococcus aureus*	51 (12.3)
MRSA	48 (11.5)
Coagulase negative staphylococci	14 (3.4)
*Streptococcus* spp.	18 (4.3)
*S. anginosus*	9 (2.2)
Anaerobes	16 (3.8)
*Bacteroides fragilis*	6 (1.4)
*Bacteroides ovatus*	1 (0.2)
*Bacteroides thetaiotaomicron*	2 (0.5)
*Clostridiodes difficile*	7 (16.8)
Fungal	2 (0.5)
*Mycobacterium* spp.	
*Mycobacterium abscessus*	14 (3.4)
Polymicrobial	157 (37.7)

^*a*^ Total may exceed *n* of 416 due to polymicrobial infections.

^*b*^ spp., species; MRSA, methicillin-resistant *S. aureus*; MSSA, methicillin-susceptible *S. aureus*.

^*c*^ Data are presented as number (%), as appropriate.

Isolate baseline MICs are displayed in Table 4. Notably, only 27 (6.5%) of isolates underwent susceptibility testing for eravacycline and of those, 88.9% (*n* = 24/27) were MIC testing and 11.1% (*n* = 3/27) were disk diffusion. Eravacycline susceptibility testing was most often conducted for *A. baumannii* (55.6%, *n* = 15/27) and carbapenem-resistant *K. pneumoniae* (22.2%, *n* = 6/27) isolates. In contrast, 49.8% (*n* = 207/416) of all isolates underwent susceptibility testing for minocycline (10%, *n* = 42/416) and/or tigecycline (46%, *n* = 191/416). The overall median (IQR) eravacycline MIC was 0.5 µg/mL (0.25, 1), MIC range was ≤0.125 to 2 µg/mL, and MIC_90_ was 1 µg/mL. For minocycline, the median (IQR) MIC was 2 µg/mL (4, 12), MIC range was 1 to 16 µg/mL, and MIC_90_ was 8 µg/mL. For tigecycline, the median (IQR) MIC was 2 µg/mL (1, 2), MIC range was 1 to 4 µg/mL, and MIC_90_ was 2 µg/mL.

**TABLE 4:** Eravacycline MIC distribution by organism^*c*^

Organism	MIC (μg/mL)^*a*^					Disk diffusion (mm)^*b*^		
	≤0.125	0.25	0.5	1	2	12	14	18
*A. baumannii*	3	0	5	3	2	1	1	0
*E. cloacae*	0	0	0	1	0	0	0	0
*E. coli*	0	0	0	0	2	0	0	0
*K. pneumoniae*	2	4	0	0	0	0	0	1
*S. maltophilia*	0	0	0	2	0	0	0	0
**Total**	5	4	5	6	4	1	1	1

^*a*^ MIC tests: Liofilchem MTS, Thermo Scientific Sensititre, and bioMerieux ETEST.

^*b*^ Disk diffusion test: HardyDisk.

^*c*^ MIC, minimum inhibitory concentration.

### Clinical outcomes and tolerability

Clinical outcomes and tolerability data are displayed in Table 5. In total, 75.7% (*n* = 315/416) of patients demonstrated clinical success. Of those, survival and absence of microbiological recurrence within 30 days of eravacycline completion occurred in 94.7% (*n* = 394/416) and 94.5% (*n* = 393/416) of patients, respectively, while 84.1% (*n* = 350/416) improved clinically within 96 h of eravacycline initiation. Most patients that did not survive to 30 days following eravacycline completion (5.3%, *n* = 22/416) had a positive sputum (40.9%, *n* = 9/22) or blood (31.8%, *n* = 7/22) culture and/or the culture was positive for an *Enterococci* spp. (31.8%, *n* = 7/22), *K. pneumoniae* (18.2%, *n* = 4/22), or S. maltophilia (18.2%, *n* = 4/22).

**TABLE 5:** Clinical outcomes and tolerability (*n* = 416)

Parameter^*a*^	Value
Clinical success^*b*^	315 (75.7)
30-day survival	394 (94.7)
Clinical improvement^*c*^	350 (84.1)
Absence of microbiological recurrence^*d*^	393 (94.5)
Microbiological recurrence	23 (5.5)
Symptomatic	19 (4.6)
Treated with antibiotic(s)	19 (4.6)
Treatment-emergent resistance	0 (0)
Hospital readmission	
30-day	77 (18.5)
60-day^*e*^	81 (23.9)
Positive culture on readmission^*f*^	10 (6.3)
ERV on readmission^*f*^	14 (8.9)
Adverse effects	39 (9.4)
Nephrotoxicity	4 (1)
Gastrointestinal intolerance^*g*^	20 (4.8)
Electrolyte disturbance	1 (0.2)
Encephalopathy	3 (0.7)
Hepatotoxicity	7 (1.7)
Dermatologic reaction	1 (0.2)
Infusion site phlebitis	2 (0.5)
Catheter site pain	1 (0.2)
ERV discontinuation secondary to an adverse effect	9/39 (23.1)
Gastrointestinal intolerance	6/9 (66.7)
Hepatotoxicity	3/9 (33.3)

^*a*^ Data are presented as number (%).

^*b*^ Clinical success: Patient survival and absence of microbiological recurrence at 30 days from the end of eravacycline therapy and clinical improvement within 96 h of eravacycline initiation.

^*c*^ Clinical improvement: The resolution of infectious signs and symptoms including infection-related abnormal white blood cell count/temperature or as documented by the physician in clinical notes.

^*d*^ Microbiological recurrence: An isolate of the same bacteria (at species level) from the same culture source taken after a negative culture.

^*e*^ Patients with a 30-day readmission were muted from 60-day readmission total.

^*f*^ Percent based on a denominator of 158, representing the number of patients readmitted within 30 and/or 60 days.

^*g*^ Gastrointestinal intolerance defined as nausea, vomiting, and/or diarrhea.

Eravacycline-related TEAEs occurred in 9.4% (*n* = 39/416) of patients with the majority (51.3%, *n* = 20/39) being gastrointestinal in nature, while the remaining TEAEs occurred in <2% of the cohort. In total, 23.1% of patients (*n* = 9/39) had eravacycline discontinued secondary to the TEAE (gastrointestinal intolerance *n* = 6, hepatotoxicity *n* = 3). For hospital readmission, 18.5% (*n* = 77/416) and 23.9% (*n* = 81/339) were readmitted within 30 and 60 days of discharge, respectively, with 6.3% (*n* = 10/158) experiencing microbiological recurrence at 30 days and 8.9% (*n* = 14/158) receiving eravacycline upon readmission. Of the patients experiencing microbiological recurrence (5.5%, *n* = 23/416), positive cultures were isolated from the respiratory tract (56.5%, *n* = 13/23), blood (17.4%, *n* = 4/23), wound (17.4%, *n* = 4/23), and urine (8.7%, *n* = 2/23) and grew *A. baumannii* (56.5%, *n* = 13/23), *S. maltophilia* (17.4%, *n* = 4/23), *E. faecium* (13%, *n* = 3/23), or *S. aureus* (13%, *n* = 3/23).

## DISCUSSION

This study provides valuable insight into the real-world use of eravacycline for the treatment of various infections in U.S. hospitals in the 4 years following its FDA approval. The data herein suggest that eravacycline is predominantly used as consolidation therapy for monomicrobial infections from a variety of sources. The broad activity and low MIC_90_ values of eravacycline against an array of Gram-negative and Gram-positive bacteria, including those demonstrating multidrug resistance, make eravacycline a therapeutic option in such challenging clinical scenarios.

These data also identified that eravacycline was commonly used as definitive therapy for infections caused by carbapenem-resistant *A. baumannii* and *Enterobacterales* spp., which make up greater than one-fifth of the study cohort. The use of eravacycline for carbapenem-resistant *Acinetobacter* spp. infections in the study cohort is particularly noteworthy since eravacycline has not yet earned an indication specifically for *Acinetobacter* spp., nor has it been assigned a CLSI or U.S. Food & Drug Administration breakpoint despite demonstrating *in vitro* activity against MDR *A. baumannii* isolates (20). Similarly, eravacycline does not have an approved indication for the treatment of respiratory or acute bacterial skin and skin structure infections, the two most common sites of positive culture attainment in this cohort (21). Additionally, compared to patients in the eravacycline IGNITE I/IV clinical trials, our study population required a significantly higher level of care. Almost half were admitted to the ICU, those on ventilators received eravacycline therapy for an average of over 3 weeks, and eravacycline was often used to treat pathogens that have historically been challenging to manage. Therefore, these findings enrich limited data suggesting that eravacycline could be a potential treatment for challenging cases, such as infections caused by CRE, carbapenem-resistant *Acinetobacter* spp., and *Stenotrophomonas maltophilia* even though recent therapeutic guidance does not currently recommend eravacycline for these conditions (22, 23). Additional data is warranted to establish the effectiveness of eravacycline in these specific patient and clinical scenarios.

The incidence of eravacycline-related adverse events and subsequent eravacycline discontinuation are like that reported in the IGNITE I/IV clinical trials with the most common being gastrointestinal disorders. While gastrointestinal disorders are more common with the tetracycline class of antibiotics, available RCT and observational data demonstrate that the incidence of drug-related gastrointestinal disorders is approximately two to five-fold lower than that reported for the other tetracyclines including omadacycline, minocycline, and tigecycline (24 – 28).

While this study is the largest report of eravacycline use in U.S. hospitals to date, it has important limitations including its retrospective, observational design, and a lack of control group to validate the role of eravacycline in reported clinical effectiveness and tolerability. Furthermore, this study highlights the limited antimicrobial susceptibility testing of eravacycline occurring in U.S. hospital-affiliated microbiology laboratories. Limited testing may be due to a lack of eravacycline breakpoints for most organisms including *Acinetobacter* and *Stenotrophomonas maltophilia* and/or limited available antimicrobial susceptibility tests for eravacycline. There are only two FDA-cleared commercial automated antimicrobial susceptibility tests (AST) with eravacycline on-panel (e.g., VITEK 2 AST Gram-Negative Panel Assay and MicroScan Neg Urine Combo 90) (29, 30), and other available AST are limited to HardyDisk, Liofilchem MTS, Thermo Scientific Sensititre, and bioMerieux ETEST (31 – 34). Unfortunately, there remains scant *in vitro* susceptibility data comparing isolate eravacycline MIC data to that of other novel and standard of care antibiotics. In the current study, approximately half of participating centers used the isolate tigecycline MIC to guide eravacycline use for *Acinetobacter* spp. and carbapenem-resistant *K. pneumoniae* infections, which may be problematic since tigecycline breakpoints are not established for eravacycline. Surveillance data of eravacycline *in vitro* activity against Gram-negative bacilli aligns with limited MIC data presented herein; however, more data are needed to elucidate the appropriateness of this practice at an organism and infectious source level.

In conclusion, eravacycline is being used in real-world clinical settings to treat a broad range of Gram-negative and Gram-positive aerobic and anaerobic bacteria in the United States, including those demonstrating multidrug-resistance, with consistently low reported drug-related TEAEs. This study adds to the growing body of evidence that supports the clinical success and tolerability of eravacycline in the treatment of complicated infections. However, the limited availability of antimicrobial susceptibility data highlights the need for continued monitoring and surveillance of antibiotic resistance patterns. Further studies are warranted to evaluate the long-term safety and efficacy of eravacycline in different patient populations and clinical settings.
